# Pre-Transplant Laboratory Parameters and Early Outcomes in HBV-Related Deceased Donor Liver Transplantation

**DOI:** 10.3390/diagnostics16203346

**Published:** 2026-10-14

**Authors:** Ayfer Serin, Safiye Koçulu Demir, İsmail Sivri, Ender Anılır, Birkan Bozkurt, Yaman Tokat

**Affiliations:** 1 Department of Gastroenterology, University of Health Sciences, Bagcilar Training and Research Hospital, Istanbul 34200, Türkiye; 2 Infectious Diseases and Clinical Microbiology, Sancaktepe Şehit Prof. Dr İlhan Varank Training and Research Hospital, Istanbul 34785, Türkiye; 3 Department of Anatomy, Faculty of Medicine, Kocaeli University, Umuttepe Campus, Kocaeli 41000, Türkiye; 4 General Surgery, Ödemiş State Hospital, İzmir 35750, Türkiye; 5 General Surgery, Başakşehir Çam, and Sakura City Hospital, Istanbul 34480, Türkiye; 6 Department of General Surgery, Acıbadem Fulya Hospital, Istanbul 34349, Türkiye

**Keywords:** deceased donor liver transplantation, laboratory parameters, biliary complications, potassium, risk assessment, prognosis

## Abstract

**Background**: Early postoperative outcomes after deceased donor liver transplantation (DDLT) are influenced by recipient, donor, and perioperative factors. Routinely available pre-transplant laboratory tests may provide complementary information for risk assessment. This study aimed to explore associations between routine pre-transplant laboratory parameters and early postoperative outcomes after DDLT. **Methods**: This retrospective single-center descriptive pilot study included 32 adult recipients who underwent DDLT for hepatitis B virus-related liver disease between 2004 and 2019. Pre-transplant biochemical and hematologic parameters were evaluated in relation to in-hospital mortality, biliary complications, infectious complications, bleeding, and hospital stay. Descriptive group comparisons and exploratory receiver operating characteristic analyses were performed. **Results**: In-hospital mortality occurred in 6/32 recipients (18.8%), biliary complications in 5/32 (15.6%), infectious complications in 9/32 (28.1%), and postoperative bleeding in 2/32 (6.2%). Routine laboratory parameters did not significantly discriminate mortality. Among 28 recipients with available potassium measurements, four developed biliary complications. Potassium showed exploratory discrimination for biliary complications (AUC = 0.818, *p* = 0.048), based on only four biliary events. Bilirubin and GGT did not significantly discriminate mortality. **Conclusions**: Routine pre-transplant laboratory parameters showed no consistent association with in-hospital mortality after DDLT. The potassium association is hypothesis-generating and requires validation in larger cohorts before clinical application.

## 1. Introduction

Liver transplantation remains the definitive treatment for patients with end-stage liver disease and selected patients with hepatocellular carcinoma (HCC), providing substantial improvements in long-term survival and quality of life. Despite major advances in surgical techniques, perioperative management, immunosuppressive therapy, and organ preservation, early postoperative complications continue to represent an important cause of morbidity, prolonged hospitalization, graft dysfunction, and mortality after transplantation. As donor and recipient characteristics have become increasingly complex, accurate pre-transplant risk assessment has become an essential component of perioperative management and individualized patient care [1,2,3].

Several prognostic models have been developed to estimate outcomes after liver transplantation. The Model for End-Stage Liver Disease (MELD), MELD-Na, and MELD 3.0 are widely used for organ allocation and assessment of disease severity but were not designed to predict early postoperative complications or short-term clinical outcomes after transplantation. Similarly, composite models incorporating donor and recipient characteristics, including the Donor Risk Index (DRI), Survival Outcomes Following Liver Transplantation (SOFT), and Balance of Risk score, provide only moderate predictive performance in clinical practice [3]. Consequently, there remains a need for simple, widely available, and clinically applicable markers that can complement existing risk assessment tools before transplantation.

Routine laboratory parameters obtained during the pre-transplant evaluation are inexpensive, universally available, and reflect multiple aspects of recipient physiology, including hepatic and renal function, metabolic reserve, nutritional status, and electrolyte balance. Several studies have reported associations between laboratory variables such as bilirubin, liver enzymes, renal function tests, electrolytes, and biochemical indicators of graft function with postoperative complications and transplant outcomes. However, the predictive performance of individual laboratory markers has generally been inconsistent, and most previous investigations have focused on postoperative biochemical changes or integrated prediction models rather than routinely available pre-transplant laboratory measurements [4,5,6,7].

Routine pre-transplant laboratory measurements may offer complementary descriptive information when considered alongside established clinical and transplant-specific factors. Whether individual measurements improve prediction of early outcomes requires evaluation in adequately powered, independently validated cohorts [4,5,6,7].

Therefore, the present study aimed to explore the associations between routinely available pre-transplant laboratory parameters and early clinical outcomes in adult recipients undergoing DDLT for hepatitis B virus (HBV)-related liver disease. The analysis focused on in-hospital mortality and major postoperative complications, with particular attention to whether simple routine measurements could provide clinically relevant hypothesis-generating signals for future validation.

## 2. Materials and Methods

### 2.1. Study Design and Patient Population

This retrospective single-center descriptive pilot study included adult patients who underwent deceased donor liver transplantation (DDLT) for hepatitis B virus (HBV)-related liver disease between 2004 and 2019 at the Liver Transplantation Center of Şişli Florence Nightingale Hospital, Demiroğlu Bilim University, Istanbul, Türkiye. The study represents a subgroup analysis of an institutional transplantation database. Only patients who underwent DDLT were included.

**Figure 1:**
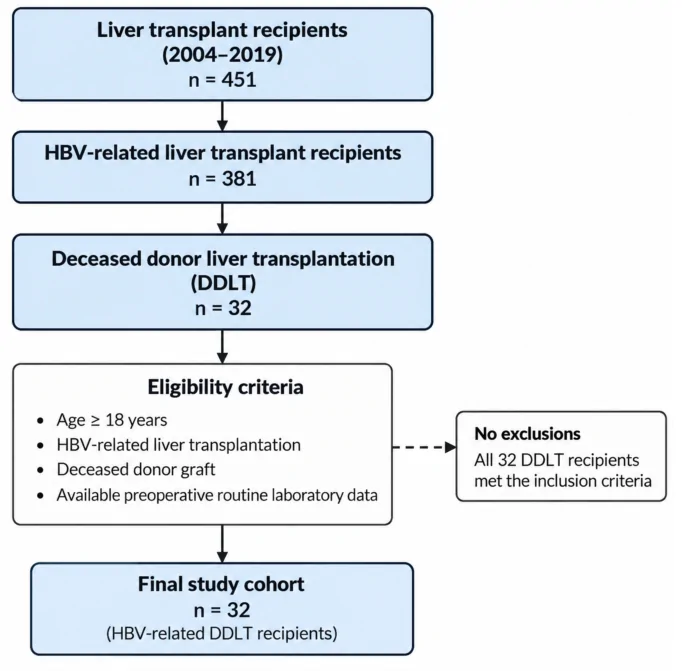
Study flow diagram. The final cohort of 32 adult recipients who underwent deceased donor liver transplantation for HBV-related liver disease. The solid arrows indicate the sequential patient selection process. The dashed arrow points to the box indicating that no patients were excluded after assessment of the eligibility criteria. The blue color is used for visual clarity and does not represent a separate clinical category.

Patients aged ≥18 years who underwent primary DDLT and had available pre-transplant clinical and laboratory data were eligible for inclusion. Patients who underwent living donor liver transplantation, retransplantation, multi-organ transplantation, or had incomplete clinical records that precluded outcome assessment were excluded. The final study cohort consisted of 32 recipients (Figure 1).

### 2.2. Ethical Approval

The study protocol was approved by the Ethics Committee of Demiroğlu Bilim University, Istanbul, Türkiye (Approval No. 2020-13-02; 21 July 2020). The study was conducted in accordance with the ethical principles of the Declaration of Helsinki.

### 2.3. Data Collection

Demographic characteristics, transplant-related variables, clinical outcomes, and laboratory findings were retrospectively retrieved from the institutional electronic medical record system.

Demographic variables included age, sex, body mass index (BMI), underlying liver disease, hepatocellular carcinoma (HCC), and diabetes mellitus.

Routine laboratory parameters obtained during the pre-transplant evaluation included the international normalized ratio (INR), aspartate aminotransferase (AST), alanine aminotransferase (ALT), alkaline phosphatase (ALP), gamma-glutamyl transferase (GGT), albumin, total bilirubin, blood urea nitrogen (BUN), serum creatinine, sodium, potassium, glucose, and glycated hemoglobin (HbA1c).

The analytical dataset did not systematically contain donor-specific characteristics, detailed operative factors, acute or chronic rejection episodes, or patient-level perioperative induction and maintenance immunosuppressive regimens. Accordingly, rejection incidence and the distribution of immunosuppressive protocols could not be determined from this dataset. HBV DNA levels, antiviral treatment details, and decompensation phenotypes were not consistently available for systematic evaluation. Missing information was not interpreted as evidence that a clinical event or treatment was absent.

Clinical outcomes included postoperative biliary complications, postoperative infectious complications, postoperative bleeding, hospital length of stay, and in-hospital mortality.

### 2.4. Study Outcomes

The primary outcome was in-hospital mortality following deceased donor liver transplantation.

Secondary outcomes included postoperative biliary complications, postoperative infectious complications, postoperative bleeding, and hospital length of stay. Hospital length of stay was defined as the interval between transplantation and hospital discharge.

### 2.5. Statistical Analysis

Continuous variables were expressed as median (interquartile range [IQR]) or mean ± standard deviation (SD), according to data distribution. Categorical variables were presented as frequencies and percentages.

Comparisons between groups were performed using the independent-samples *t* test or the Mann–Whitney *U* test for continuous variables and the chi-square test or Fisher’s exact test for categorical variables, as appropriate.

Because the endpoint was in-hospital mortality and discharge times varied, mortality was reported as a count and proportion rather than analyzed using Kaplan–Meier methods. All analyses used available observations without imputation. Exploratory laboratory comparisons involved multiple tests; *p* values were not adjusted for multiplicity and should not be interpreted as confirmatory evidence.

Receiver operating characteristic (ROC) curve analysis was used only for exploratory description of selected laboratory–outcome associations. AUC estimates and unadjusted *p* values are reported with caution because of sparse events and multiple comparisons. Post hoc thresholds were not emphasized because the analysis was exploratory and event counts were sparse. Analyses used available observations for each variable; no imputation was performed.

Conventional MELD and MELD-Na scores were retrospectively reconstructed for deceased-donor recipients only, using available laboratory measurements recorded as pre-transplant in the source dataset. Conventional MELD used bilirubin, INR, and creatinine (component values below 1 set to 1; creatinine capped at 4 mg/dL). MELD-Na used serum sodium restricted to 125–137 mmol/L and MELD restricted to 6–40. Scores were rounded to the nearest integer. Scores were calculable for 31 and 28 patients, respectively. The original timing of individual laboratory draws relative to transplantation could not be independently verified for every record; this retrospective limitation should be considered when interpreting the scores.

All statistical analyses were performed using SPSS version 14.0 (SPSS Inc., Chicago, IL, USA). All tests were two-sided, and a *p* value < 0.05 was considered statistically significant.

Generative AI was not used. The authors independently reviewed and approved all analyses, interpretations, references, and the final manuscript.

## 3. Results

A total of 32 adult patients who underwent deceased donor liver transplantation (DDLT) were included in the study. Baseline demographic, clinical, and pre-transplant laboratory characteristics are summarized in Table 1. The availability of selected baseline measurements varied: age 24/32 (75.0%; 25.0% missing), MELD 31/32 (96.9%; 3.1% missing), MELD-Na 28/32 (87.5%; 12.5% missing), potassium and sodium each 28/32 (87.5%; 12.5% missing), and HbA1c 22/32 (68.8%; 31.2% missing).

The annual distribution of transplantations was as follows: 2007 (*n* = 1), 2011 (*n* = 2), 2013 (*n* = 14), 2014 (*n* = 1), 2015 (*n* = 5), 2016 (*n* = 5), 2017 (*n* = 2), and 2018 (*n* = 2). Three transplantations occurred during 2004–2011 and 29 during 2012–2019. Given the markedly unequal era-specific sample sizes and sparse outcome events, formal early-versus-late comparisons were not performed.

Continuous variables are presented as median (interquartile range [IQR]); categorical variables as *n* (%). Available sample sizes are indicated in the Overall column. *p* values were calculated using the Mann–Whitney *U* test for continuous variables and Fisher’s exact test for categorical variables, as appropriate. MELD and MELD-Na were retrospectively calculated from available pre-transplant laboratory values; their calculations were checked against the study records by the investigators.

Among 24 recipients with recorded age, the median was 58.0 years (IQR, 46.8–61.3), and 27 patients (84.4%) were male. Hepatocellular carcinoma (HCC) was present in 10 patients (31.2%), whereas diabetes mellitus was identified in six patients (18.8%). During hospitalization, postoperative infectious complications occurred in nine patients (28.1%), postoperative biliary complications in five (15.6%), postoperative bleeding in two (6.2%), and in-hospital mortality in six patients (18.8%). The study dataset comprised 20 recipients categorized as HBV, 10 as HBV-associated HCC, and two as HBV–HDV coinfection; these are dataset categories rather than a complete characterization of viral replication or treatment status. Age was available for 24/32 (75.0%), INR for 31/32 (96.9%), sodium and potassium each for 28/32 (87.5%), and HbA1c for 22/32 (68.8%).

Early biliary complications were recorded in five recipients (15.6%). Available notes described strictures in two recipients and reoperation for a fistula in one, although a biliary origin of the fistula could not be confirmed. Complication subtype was undocumented in two cases; exact event dates and anastomotic status were not reliably available. These descriptive categories should therefore be interpreted cautiously.

No statistically significant differences were detected in the available demographic or pre-transplant laboratory comparisons between survivors and non-survivors (all *p* > 0.05). Reconstructed MELD scores were available for 31 recipients (survivors: *n* = 25; non-survivors: *n* = 6), with medians (IQR) of 14.0 (10.0–18.0) and 14.5 (11.5–21.25), respectively (Mann–Whitney *U* *p* = 0.861). MELD-Na scores were available for 28 recipients (survivors: *n* = 23; non-survivors: *n* = 5), with medians (IQR) of 17.0 (10.5–19.5) and 18.0 (11.0–25.0), respectively (*p* = 0.718). These comparisons were exploratory and did not show statistically significant differences.

**Figure 2:**
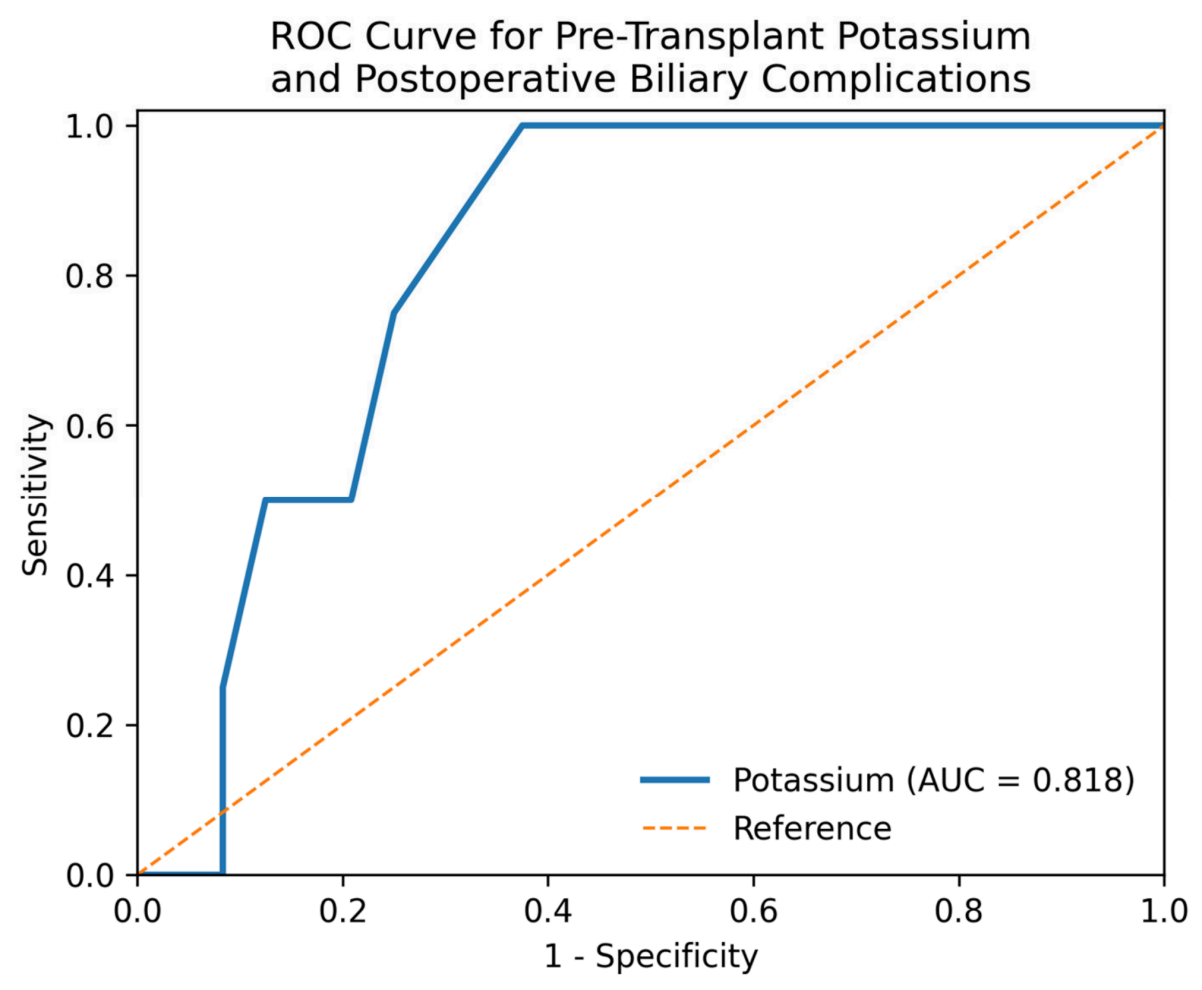
Exploratory receiver operating characteristic (ROC) curve for pre-transplant serum potassium and recorded postoperative biliary complications (*n* = 28; four biliary events). The curve is hypothesis-generating and should not be used to derive a clinical decision threshold.

Exploratory ROC analysis of selected laboratory parameters showed no consistent discrimination for in-hospital mortality (Table 2). Total bilirubin (AUC = 0.442, *p* = 0.681) and GGT (AUC = 0.429, *p* = 0.612) were not significantly associated with mortality. Among 28 recipients with available pre-transplant potassium values, only four developed biliary complications. Potassium yielded an exploratory AUC of 0.818 (unadjusted *p* = 0.048; Figure 2). Given the small event count and the numerous exploratory comparisons, this result may reflect chance and should not be interpreted as a clinically useful predictor.

All laboratory variables represent available pre-transplant measurements. Potassium was measured in 28 recipients, including four who developed biliary complications. AUC, area under the receiver operating characteristic curve. All *p* values are unadjusted; results are exploratory in view of the small sample and multiple comparisons. Post hoc cutoff values and associated threshold-dependent performance estimates are not presented as clinically validated results.

## 4. Discussion

The present exploratory study evaluated routinely available pre-transplant laboratory parameters in adult recipients undergoing DDLT for HBV-related liver disease. The main finding was that individual routine laboratory measurements showed limited discrimination for in-hospital mortality. In contrast, pre-transplant serum potassium showed an exploratory association with postoperative biliary complications. Given the small number of biliary events, this observation should be viewed as a hypothesis-generating signal rather than evidence for a clinically actionable cutoff.

Accurate pre-transplant risk stratification remains a major challenge in liver transplantation. While the MELD score and its subsequent modifications have substantially improved organ allocation and estimation of disease severity, they were not designed to predict early postoperative complications after transplantation. Likewise, donor–recipient composite models, including the Donor Risk Index (DRI), Survival Outcomes Following Liver Transplantation (SOFT), and Balance of Risk score, provide only moderate discrimination for short-term postoperative outcomes. Consequently, there is increasing interest in identifying simple, inexpensive, and widely available biomarkers that may improve perioperative risk assessment when integrated with established clinical prediction models [2,3,8,9,10].

In the present study, neither pre-transplant total bilirubin nor gamma-glutamyl transferase (GGT) significantly predicted in-hospital mortality. Both biomarkers are routinely used to assess hepatocellular injury and cholestatic dysfunction; however, their prognostic value has varied considerably among published studies. Bilirubin concentrations are influenced by multiple factors, including the severity of chronic liver disease, biliary obstruction, hemolysis, and nutritional status, whereas GGT reflects cholestatic injury and oxidative stress but lacks specificity for postoperative outcomes. Previous investigations have demonstrated stronger associations between perioperative bilirubin dynamics and adverse graft outcomes, although these studies primarily evaluated serial postoperative measurements rather than isolated pre-transplant laboratory values. Therefore, direct comparison with our findings should be interpreted cautiously [5,6,11,12,13,14].

The observed association between pre-transplant potassium and postoperative biliary complications was unexpected and based on only four events among 28 recipients with potassium measurements. No established biological mechanism or independent clinical evidence supports potassium as a biliary complication biomarker. The exploratory AUC and unadjusted *p* value should therefore be interpreted cautiously, particularly given the multiple laboratory and outcome comparisons. The finding is hypothesis-generating and does not establish causation or a clinically validated cutoff. Contemporary meta-analyses emphasize graft, anatomical, surgical, and biliary-reconstruction factors when assessing biliary complications, rather than serum potassium alone [15,16,17,18].

The absence of strong laboratory discrimination for mortality is clinically plausible. Early outcomes after liver transplantation reflect complex interactions among recipient condition, donor quality, ischemia–reperfusion injury, operative complexity, perioperative hemodynamics, immunosuppression, and postoperative complications. A cohort of 32 recipients with six in-hospital deaths is therefore unlikely to support a stable multivariable prediction model. For this reason, the present analysis emphasizes descriptive and exploratory discrimination rather than construction of a new prognostic score. The established literature on postreperfusion syndrome, extended-criteria donors, and early allograft dysfunction illustrates additional determinants of early post-transplant outcomes not assessed here [19,20,21].

The strengths of the present study include the relatively homogeneous cohort of HBV-related DDLT recipients and the evaluation of inexpensive routine laboratory measurements already obtained during standard pre-transplant assessment. The analysis also considered several clinically relevant early outcomes, allowing a focused assessment of whether readily available laboratory information contains signals that may warrant validation in larger cohorts.

Published studies of HBV-related liver transplantation describe improved outcomes in the era of effective hepatitis B immunoglobulin and nucleos(t)ide analogue prophylaxis [22,23]. The literature also addresses the relationship between HBV prophylaxis and hepatocellular carcinoma recurrence [24]. In our historical cohort, in-hospital mortality occurred in 6/32 recipients (18.8%), infections in 9/32 (28.1%), and biliary complications in 5/32 (15.6%). These early hospital-based outcomes are not directly comparable with published long-term survival or recurrence endpoints. Differences in recipient and donor characteristics, transplant era, antiviral treatment, follow-up duration, and outcome definitions further limit comparisons. These findings should be interpreted as descriptive and hypothesis-generating.

Several limitations should be acknowledged. This retrospective, single-center historical cohort comprised only 32 recipients, including six in-hospital deaths, five biliary complications, nine infections, and two bleeding events; these event counts preclude reliable prognostic modeling. Missing laboratory measurements reduced effective sample sizes and may introduce selection bias and unstable estimates. MELD and MELD-Na were retrospectively calculated from available pre-transplant laboratory measurements and checked against the study records; comparisons remain exploratory given the limited sample size. Changes in transplant practice across the recruitment period may have introduced additional heterogeneity, and no reliable transplant-era effect could be estimated in this small cohort. Donor age, ischemia times, graft steatosis, preservation technique, biliary reconstruction, blood loss, transfusion requirements, vasopressor use, and early allograft dysfunction were not systematically captured in the analytical dataset. Acute and chronic rejection episodes and induction and maintenance immunosuppressive regimens were not systematically recorded, so rejection incidence and treatment-related contributions to outcomes could not be assessed. Detailed HBV DNA, antiviral management, and decompensation phenotypes were unavailable for systematic analysis. Incomplete characterization and timing of biliary complications further limit interpretation. No causal inference or clinically actionable risk stratification is justified. The observed association arose amid multiple exploratory laboratory–outcome comparisons, without multiplicity adjustment, and may represent a chance finding. The MELD and MELD-Na calculations were checked against available study records; their clinical interpretation still depends on the provenance and timing of pre-transplant laboratory sampling.

Postoperative infectious complications remain an important cause of early morbidity after liver transplantation. In the present cohort, infections occurred in 28.1% of recipients. The study was not powered to establish a laboratory-based infection prediction model, reinforcing the need for larger datasets if routine pre-transplant tests are to be incorporated into multidimensional risk assessment strategies.

From a clinical perspective, routine pre-transplant laboratory biomarkers should be regarded as complementary rather than stand-alone prognostic tools. More comprehensive approaches integrate recipient characteristics, donor variables, operative factors, postoperative parameters, and validated clinical models [9,21,25]. Advanced approaches, including machine-learning techniques, may facilitate multidimensional prediction in sufficiently large, well-characterized datasets [26,27]. However, our small cohort, sparse events, and incomplete donor and perioperative information preclude meaningful development or validation of such models. The observed laboratory associations therefore remain descriptive and hypothesis-generating. The recent multicenter and systematic-review literature supports studying multivariable approaches incorporating donor, recipient, and perioperative characteristics, but these models cannot be fitted reliably in the present small cohort [26,28,29,30,31].

## 5. Conclusions

In conclusion, this small historical descriptive pilot cohort did not demonstrate consistent associations between routine pre-transplant laboratory measurements and in-hospital mortality after HBV-related DDLT. The potassium–biliary complication association was based on very few events and remains an exploratory, hypothesis-generating observation. The findings do not support clinical risk stratification or a validated laboratory cutoff and require assessment in larger contemporary cohorts incorporating donor and perioperative variables.

## Figures and Tables

**Table 1:** Baseline demographic, clinical, pre-transplant laboratory, and postoperative outcome characteristics according to in-hospital mortality.

Variable	Overall (*n* = 32)	Survivors (*n* = 26)	Non-Survivors (*n* = 6)	*p* Value
Age, years	58.00 (46.75–61.25) [*n* = 24]	55.00 (46.50–61.00)	59.00 (58.00–61.00)	0.748
INR	1.40 (1.27–1.69) [*n* = 31]	1.40 (1.32–1.63)	1.39 (1.21–1.95)	0.900
MELD score	14.0 (10.5–18.5) [*n* = 31]	14.0 (10.0–18.0) [*n* = 25]	14.5 (11.5–21.25) [*n* = 6]	0.861
MELD-Na score	17.0 (10.75–20.5) [*n* = 28]	17.0 (10.5–19.5) [*n* = 23]	18.0 (11.0–25.0) [*n* = 5]	0.718
AST, U/L	84.50 (43.50–133.75) [*n* = 30]	98.00 (51.00–164.00)	51.00 (40.00–76.00)	0.191
ALT, U/L	58.50 (30.00–111.75) [*n* = 30]	50.00 (30.00–96.25)	87.00 (60.75–123.00)	0.203
ALP, U/L	139.00 (105.00–205.00) [*n* = 29]	136.50 (108.75–207.25)	139.00 (98.00–175.00)	0.674
GGT, U/L	84.50 (45.50–129.25) [*n* = 32]	104.50 (48.50–137.75)	68.50 (51.25–82.75)	0.612
Albumin, g/dL	3.10 (2.73–3.68) [*n* = 30]	3.10 (2.90–3.78)	2.70 (2.10–3.30)	0.190
Total bilirubin, mg/dL	2.25 (0.97–4.72) [*n* = 32]	2.25 (1.19–4.52)	1.75 (0.77–5.23)	0.681
BUN, mg/dL	13.00 (11.00–20.00) [*n* = 32]	13.00 (11.00–19.25)	16.50 (13.50–21.00)	0.275
Creatinine, mg/dL	0.72 (0.63–1.00) [*n* = 32]	0.78 (0.66–1.00)	0.70 (0.62–1.22)	0.961
Sodium, mmol/L	137.00 (134.00–140.00) [*n* = 28]	137.00 (135.00–140.00)	136.00 (133.00–138.00)	0.487
Potassium, mmol/L	4.10 (3.98–4.33) [*n* = 28]	4.10 (3.95–4.30)	4.10 (4.00–4.60)	0.567
Glucose, mg/dL	101.00 (96.00–120.00) [*n* = 29]	104.50 (93.75–120.75)	100.00 (98.00–120.00)	0.583
HbA1c, %	5.35 (4.88–6.65) [*n* = 22]	5.35 (4.88–6.65)	5.65 (5.05–6.62)	0.898
Hospital stay, days	14.50 (9.75–20.00) [*n* = 32]	16.00 (10.00–20.00)	11.50 (8.75–16.50)	0.438
Male sex	27 (84.4)	21 (80.8)	6 (100.0)	0.555
HCC	10 (31.2)	7 (26.9)	3 (50.0)	0.346
Diabetes mellitus	6 (18.8)	4 (15.4)	2 (33.3)	0.310
Biliary complications	5 (15.6)	4 (15.4)	1 (16.7)	1.000
Postoperative bleeding	2 (6.2)	1 (3.8)	1 (16.7)	0.345
Postoperative infection	9 (28.1)	7 (26.9)	2 (33.3)	1.000

**Table 2:** Exploratory receiver operating characteristic (ROC) analyses of pre-transplant laboratory parameters. These results are descriptive and not clinically validated.

Outcome	Predictor	*n*	AUC	*p* Value
In-hospital mortality	Total bilirubin, mg/dL	32	0.442	0.681
In-hospital mortality	GGT, U/L	32	0.429	0.612
Postoperative biliary complications	Potassium, mmol/L	28	0.818	0.048

## Data Availability

The original contributions presented in this study are included in the article. Further inquiries can be directed to the corresponding author.
